# Alkaline Pretreatment of Sugarcane Bagasse and Filter Mud Codigested to Improve Biomethane Production

**DOI:** 10.1155/2016/8650597

**Published:** 2016-09-21

**Authors:** Zahir Talha, Weimin Ding, Esmaeil Mehryar, Muhammad Hassan, Jinhua Bi

**Affiliations:** ^1^College of Engineering, Nanjing Agricultural University, Nanjing, Jiangsu 210031, China; ^2^Institute of Agricultural Resources and Environment, Jiangsu Academy of Agricultural Science, Nanjing, Jiangsu 210014, China

## Abstract

To enhance the codigestion of degradation and improve biomethane production potential, sugarcane bagasse and filter mud were pretreated by sodium hydroxide NaOH 1 N at 100°C for 15, 30, and 45 minutes, respectively. Biomethane generation from 1-liter batch reactor was studied at mesophilic temperature (37 ± 1)°C, solid concentrations of 6%, and five levels of mixing proportion with and without pretreatment. The results demonstrate that codigestion of filter mud with bagasse produces more biomethane than fermentation of filter mud as single substrate; even codigested substrate composition presented a better balance of nutrients (C/N ratio of 24.70) when codigestion ratio between filter mud and bagasse was 25 : 75 in comparison to filter mud as single substrate (C/N ratio 9.68). All the pretreatments tested led to solubilization of the organic matter, with a maximum lignin reduction of 86.27% and cumulative yield of biomethane (195.8 mL·gVS^−1^, digestion of pretreated bagasse as single substrate) obtained after 45 minutes of cooking by NaOH 1 N at 100°C. Under this pretreatment condition, significant increase in cumulative methane yield was observed (126.2 mL·gVS^−1^) at codigestion ratio of 25 : 75 between filter mud and bagasse by increase of 81.20% from untreated composition.

## 1. Introduction 

Energy plays an important role in the national security of any given country as a fuel to power the economic engine. It is convenient to use oil, coal, and natural gas for our energy needs, but they are limited and by continuous and rapid use they will run out. Furthermore, they increase greenhouse gases emission into atmosphere which causes the trapping of sun's heat and contributing to global warming [1, 2]. Ruppert has claimed that, to produce one calorie of food in the industrial world, we need to invest ten calories of oil and gas energy in the forms of fertilizer, pesticide, packaging, transportation, and running farm equipment [3].

The crisis of huge energy demand has generated more interest in the use of biomass as a potential renewable energy source that could replace fossil energy [4]. Considerable amount of waste byproduct materials is being generated through agricultural practices, mainly from various agrobased industries. Agroindustrial biomass such as sugar industry waste is an inexpensive, renewable, abundant, and rich-in-energy potential. Unfortunately, much of the biomass is often disposed of by burning, which is not restricted to developing countries alone [5].

Sugarcane bagasse is a plentiful byproduct obtained from the sugar industry, a lignocellulosic, residual material derived after the extraction of cane juice which corresponds to about 25% of the total processed sugarcane [6]. It is almost completely burnt by sugar factories themselves as fuel for boilers [7]. Recently, more efforts have been directed toward more efficient utilization of sugarcane bagasse as a raw material for pulp and paper production, boards, animal feed, and products based on fermentation [8]. Like most agricultural residues, bagasse is rich in soluble sugar, cellulose, hemicellulose, and lignin, which promotes research capabilities on bioconversion processes of this material for the production of bioethanol, biogas, and other bioproducts [9]. Therefore, anaerobic digestion of sugarcane bagasse may improve its value as well as solve contamination problem [10].

Filter mud which is also called filter-cake is a solid residue obtained from sugarcane juice before crystallization of sugar. The availability of filter mud is about 3–7% of total crushed cane [11], while the chemical composition depends on many factors including the cane variety, soil condition, nutrients applied in the field, process of clarification adopted, and other environmental factors [1]. Moreover, in some sugar industries, filter mud is disposed as garbage, or used as fertilizer, whether as raw material or by converting it into compost [12]. The feature behavior of filter mud indicates that it is highly suitable for energy production [13, 14]. It consists of hydrocarbon, sugar, and other components, and it has a good proportion of carbon to nitrogen ratio (C/N) of approximately 10–20; these are considered as significantly attractive features for generation of bioenergy by anaerobic fermentation [15–17].

Lignocelluloses consist of three major components including cellulose, hemicellulose, and lignin. Among these constituents, cellulose and hemicelluloses are polymers of sugars and can be hydrolyzed. On the other hand, lignin forms a protective covering that limits cellulose and hemicelluloses biodegradability [18]. To improve lignocelluloses material digestibility, previous studies have focused on codigestion with another material in different proportions and/or pretreatment through various methods including mechanical, thermal, chemical, and combined measures [19, 20].

To improve the yield of biomass utilization, sugarcane bagasse has been used for biofuel production (bioethanol and biogas) by high solids fed batch saccharification and fermentation process for ethanol production, and, after evaporation, the residual obtained was used for methane production through anaerobic digestion [21]. Li et al. [10] have investigated the biogas generation from sugarcane bagasse after liquid hot water (LHW) pretreatment and NaOH pretreatment; they showed that the gas yield of each pretreatment group increased significantly when compared to nonpretreated group. Pretreatment by per acetic acid (PAA) under mild conditions greatly increased the enzymatic digestibility of sugarcane bagasse and the yield of reducing sugars reached 92.04% by enzymatic hydrolysis [22].

As in previous study, thermoalkaline pretreatment of filter mud (100°C, Ca(OH)_2_) for different pretreatment times and lime loading resulted in an excess of 72% of methane yield [12]. Codigestion of sugarcane filter mud with bagasse was investigated for anaerobic digestion in a semicontinuous feeding to assess the main parameters used for large scale digesters [23]. Generation of biogas was increased significantly after mixing press mud in different proportion with sugarcane bagasse and other substrates [15].

Among all the pretreatment methods, alkaline pretreatment has widely been studied. The main advantages of the process are concluded as follows: it is efficient in removal of lignin and it gives higher yields of reducing sugars. Although lime and other hydroxides are inexpensive, pretreatment processes have a significant impact on the configuration, efficiency, and cost of biogas production. Anaerobic digestion process has milder requirements for the pretreatment of lignocellulosic biomass. For example, anaerobic digestion microbes have higher tolerances to inhibitory compounds generated in pretreatment so that detoxification is not needed. Therefore, pretreatment might be more technoeconomically feasible for incorporation into commercial anaerobic digestion [24]. Sodium hydroxide pretreatment has a long history of study as it was used in an attempt to increase the digestibility of cellulose by rumen animals. This strong base solubilizes hemicellulose and lignin significantly under certain conditions [25]. The effectiveness of pretreatment using sodium hydroxide has showed a greater degree of enzyme hydrolysis than with other alkaline pretreatments, and it has been extensively studied to improve biogas yield from lignocellulosic biomass [24, 26] such as rice straw [27–29], corn stover [30, 31], and sugarcane bagasse [22, 32, 33]. The objectives of the present study are (1) to examine the biogas production by codigestion of sugarcane bagasse and filter mud for different mixing ratios, (2) to verify the potential of NaOH pretreatment at 100°C, and (3) to enhance the anaerobic digestion regarding methane yield augmentation. The effects of alkaline pretreatment time on lignin removal, chemical oxygen demand (COD) solubilization, and ammonia (NH_3_-N) removal have been determined for different experimental conditions.

## 2. Materials and Methods

### 2.1. Substrate

In this work, experiments are carried out using fresh filter mud and bagasse which were provided from the Zhanjiang Huazi Land-Reclamation Sugar Industry Co. Ltd. (Guangdong, Zhanjiang, China) during 2015 harvest season. Filter mud was air-dried, milled, and sieved to a particle size of less than 2 mm. It was subsequently stored in plastic bags at 4°C until use. Bagasse was first dried by the air then by oven drying in 45°C for 48 hr. after that milled with a grinder and sieved to pass 5 mm sieve, and stored in plastic bags at vented room for further analysis and fermentation.

### 2.2. Pretreatment Process

The pretreatment process was conducted on a 2-liter glass flask, substrate was weighted and placed into the flask, and one-liter NaOH 1 N was added (solid to liquid ratio was 1 : 12). For thermal treatment, autoclave (Jiang Yin Bing Jiang Medical Equipment Co. Ltd.) was used. The flask was sealed with aluminum foil and autoclaved at 100°C and 1 bar pressure for 15 min, 30 min, and 45 min, respectively, similar to work of [34, 35]. After alkali pretreatment, the substrate was washed with 500 mL pure water, then dried with oven 45°C for 48 hr, and stored in sealed bags for further analysis and anaerobic fermentations.

### 2.3. Batch Anaerobic Digestion

#### 2.3.1. Experiment Setting

The batch anaerobic digestion equipment of this study consists of two 1-liter bottles, one being used as digester and another one as water bottle for collecting biogas, a set of beakers for collecting water, connection piping components, and water bath vessel for maintaining the temperature. Each digester covered with a cap contained two circular holes, one of which acted as an opening to withdraw sludge sample to analyze the process parameters during anaerobic digestion while the second hole was connected with the water bottle through a pipe having port for taking gas sample for GC analysis. The water bottle was filled in sodium hydrogen bicarbonate to prevent CO_2_ solubility in the water. The produced biogas from each digester was captured in the water bottle and the displaced liquid was in turn collected in the set of beakers to measure the biogas volume by the water displacement technique. Biogas composition and total biogas production was measured on daily basis while pH, COD, and NH_3_-N were done every 3 days.

#### 2.3.2. Substrate Composition

Five proportions of bagasse and filter mud (basis TS total solid) were selected as shown in Table 1.

The proportion of the bagasse and filter mud was diluted to 6% total solid concentrations. It was inoculated with the sludge collected from an anaerobic digestion plant for pig farm waste (Nanjing Kaiping Family Farm, Poukou, Nanjing, China) and sieved through 20-mesh filter screen. Chemical characteristics of the sludge were determined as total solids (TS) (2.01 ± 0.05)%; volatile solids (VS) (47.90 ± 0.7)%; pH value 7.76; soluble chemical oxygen demands (CODs) 1320 mg/L; and ammonia nitrogen (NH_3_-N) 1197 mg/L.

Inoculum sludge was adjusted to be 40% from the total solid of the substrate volume; the total substrate in the digester was 0.8 liter including the sludge. pH during anaerobic digestion process was controlled to be between 6.5 and 8.5 by injecting hydrochloric acid (HCl) or sodium hydroxide (NaOH) solution into the digester through sampling hole if it was below or above the range. All anaerobic digestion tests were carried out at (37 ± 1)°C for about 35 days.

### 2.4. Analytical Methods

Total solids (TS), volatile solids (VS), ashes, total organic carbon (TOC), total nitrogen (TN), total phosphorus (TP), total potassium (TK), and soluble chemical oxygen demand (CODs) were determined according to standard methods [36]. Lignocellulosic characteristics were determined according to the Van Soest method [37], with a fiber extractor (VELP Scientifica Company, Italy). It is based on sequential extraction under neutral and acid detergent (NDF, ADF), followed by strong acid extraction (ADL). Different fractions are (a) soluble in neutral detergent fraction (1-NDF); (b) hemicelluloses (NDF–ADF) which is extracted by acid detergent; (c) cellulose (ADF–ADL) which is extracted by 76% sulphuric acid; and (d) lignin (ADL). pH was monitored in samples using digital pH meter (FE20K, Mettler-Toledo, Switzerland) capable of measuring in liquid substrates. Samples for analysis CODs and ammonia nitrogen (NH_3_-N) were centrifuged at 10,000 rpm for 4 min in a centrifuge. After centrifugation, only the supernatant was used. Ammonia nitrogen (NH_3_-N) was determined from filtered samples, which diluted with deionized water in a proportion of 1 : 200 using a bench top spectrophotometer (Lianhua Co., Shanghai).

The biogas composition (CH_4_ and CO_2_) measurement was conducted through biogas sampling from reactors by a special syringe and injection to the thermal conductivity detector (TCD) of gas chromatograph (Agilent 7820A) equipped with PQ 80–100 mesh column. The operation condition was as follows: 25 mL/min helium as the carrier gas and detector temperature 250°C and 90°C of column temperature.

### 2.5. Statistical Analyses

Two-way ANOVA (Analysis of Variance) was used to test significant differences in mean values between treated and untreated samples for lignocelluloses properties and methane production. Furthermore, the graphical representation of the data was provided by using Analytical Software Package (Graph Pad, Prism 6.01).

## 3. Results and Discussion

### 3.1. Characteristics and Composition

General characteristics of fresh sugarcane bagasse and filter mud used during batch experiments are presented in Table 2. The total solid in the samples varied from 58.9% for bagasse and 47.85% for the filter mud. Filter mud and bagasse were close in nitrogen content, while bagasse contained a considerable amount of organic carbon and organic matter concentrations.

Filter mud had greater amount of ash. The carbon to nitrogen ratio (C/N) needed for effective digestion is between 10 and 30 [38]. The C/N ratio of filter-cake is approximately 9.6 but for bagasse it is approximately 29.6. This composition mainly for bagasse is in good agreement with that reported in previous studies [6, 15].

### 3.2. Effect of Sodium Hydroxide Pretreatments on Sugarcane Bagasse Composition

The main advantages of the alkali pretreatment are removal of lignin and increasing the availability of cellulose for the bacterial metabolism during the anaerobic digestion process [39]. Lignin removal is an important part of the pretreatment process, because lignin can effectively inhibit/prevent the cellulase enzymes from hydrolyzing the cellulose. Alkaline pretreatment by adding NaOH solution causes a swelling of the biomass, which increases the internal surface area of the lignocellulose particles, as well as weakening the structural integrity of the lignocellulose and breaking bond linkages between lignin and the other carbohydrates (cellulose and hemicellulose), resulting in greater accessibility and digestibility of the cellulose fraction, and it can be depolymerized into fermentable sugars [26]. In order to determine the efficiency of different biomass plants degradation hemicellulose, cellulose and lignin content in the pretreated bagasse were measured and illustrated in Table 3.

**Figure 1:**
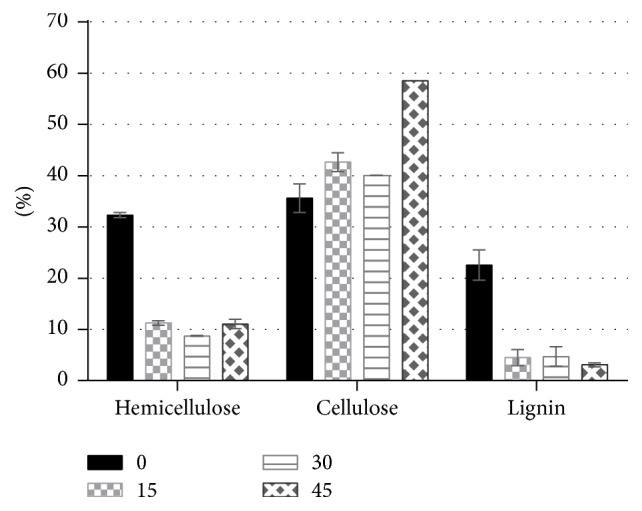
Comparison of chemical composition in bagasse after delignification by NaOH 1 N.

As shown in Table 3, untreated bagasse contained 35.61% of cellulose and 22.56% of lignin, after being autoclaved at 100°C by NaOH 1 N for 15 minutes of delignification. The cellulose increased by 16.5% and lignin decreased by 80%. Meanwhile, for 30 minutes of delignification, cellulose shifted to 40.06% or increased 11% and lignin content shifted to 4.72% or decreased 79%. Moreover, after delignification by NaOH 1 N for 45 minutes, cellulose increased by 39.1% and lignin content decreased by 86.2%. From this data, it could be observed that the cellulose content increased more and lignin content decreased by increasing the delignification time. The significant increase of cellulose content was probably because cellulose in solid phase is high and only slight fraction of lignin is extracted in the liquid phase. After pretreatment, the cellulose could be still in solid phase and small fraction will be in liquid phase. This is in agreement with the findings of Maryana et al., who evaluate the effect of alkaline pretreatment on the chemical composition and structure of sugarcane bagasse by using 1 and 2 N NaOH concentration and 4 periods of delignification and found that there was about 59.1% and 42.3% lignin loss after 1 and 2 N alkaline pretreatment in 30 and 40 minutes of delignification, respectively [34]. Wang et al. reported that the loss of hemicellulose and lignin of rice straw was 89.45% and 88.92%, respectively, at 4.0% NaOH [40]. Effect of different alkaline dosages (4% and 10% gNaOH/gTS), temperatures (40°C and 55°C), and contact times (12 h and 24 h) in structural feature of sorghum forage was investigated and it was found that, by increasing the NaOH dosage, a reduction of hemicelluloses (from 37% to 70%) and lignin contents (from 26% to 70%) was observed [41]. Therefore, it can be concluded that delignification by NaOH 1 N for 45 minutes was the most effective for delignification process because the decreasing rate of lignin level was the highest. Comparison of the level of lignin, hemicellulose, and cellulose content after being treated by NaOH 1 N is illustrated in Figure 1. It is shown that the lignin content after cooking for 45 minutes was the lowest, 3.096%. In addition, percentages of the lignin content after cooking times of 30 minutes and 15 minutes and untreated bagasse were 4.715, 4.514, and 22.563%, respectively.

### 3.3. Daily Biomethane Production

**Figure 2:**
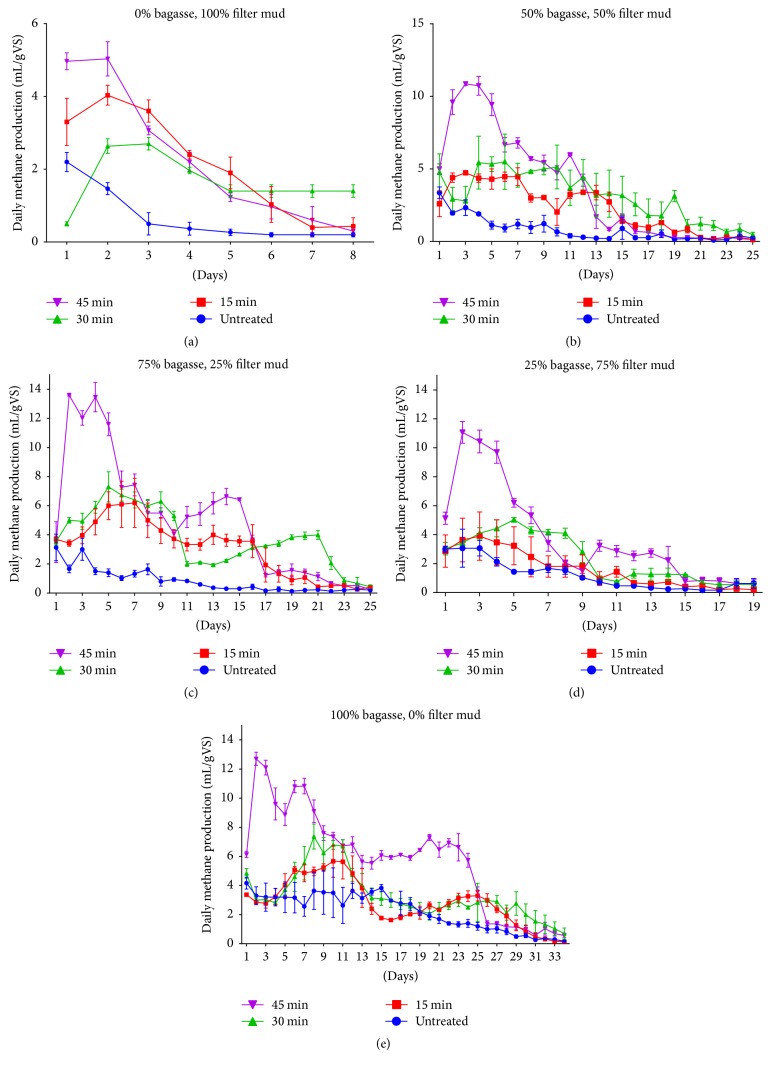
Daily methane production by different codigestion rates and pretreatments time.

In general, the daily biomethane production has been summarized in Figures 2(a)–2(e); all groups had two methane production periods. Obviously, there were higher peaks of methane production in the first 6 days, which could be mainly caused by degradation of soluble sugar in the substrate. In the second period, methane production rate decreased and goes in stability for about 10 days with the decomposition of cellulose and hemicelluloses. Furthermore, biogas production decreased slowly in the last 10 days.

#### 3.3.1. Codigestion of Filter Mud with Bagasse in C/N Ratio

Filter mud was codigested with bagasse at mesophilic temperatures (37°C) using five different combinations in order to determine the effect of codigestion on biogas and methane production. The C/N ratio of filter mud was 9.6, which is lower than the optimum required for biomethanation [42]. To increase the C/N ratio, filter mud was mixed with bagasse in different proportions as in Table 1. The overall maximum biomethane yield was 22.2 mL·gVS^−1^ (72.5% more than the digestion of pure filter mud) and was achieved in codigestion ratio of 25% filter mud and 75% bagasse with C/N ration 24.7 which is 60% greater than C/N ratio of pure filter mud. The lowest yield was obtained with filter mud as the only substrate (C/N ratio of 9.862). These results confirm that microorganism's metabolic activity is significantly influenced by the nutrient ratio [43], maximum yield occurred around a C/N ratio of 25 g C/g N, and this occurs because microorganisms utilize carbon 25 times faster than nitrogen. However, the optimum C/N ratios have been reported in literature. For anaerobic digestion of filter mud, C/N ratio of 18 was found to be optimum for biogasification of filter mud [15]. For methane formation from agroindustrial waste such as molasses, Iqbal et al. found that C/N ratio of 30, based on organic carbon and total nitrogen, is optimal [44].

The results demonstrate that codigestion of bagasse with filter mud produces less biogas than digestion of bagasse alone (76.0 and 6.1 mL·gVS^−1^ for pure bagasse and filter mud, resp.). It can be explained by the high ash content found in filter mud due to its characteristics which depend on cane variety, soil condition, and other environmental factors. This sample of filter mud was collected from Land-Reclamation Sugar Industry Company.

#### 3.3.2. Effect of Alkaline Pretreatment on Methane Yield

The methane yield is defined as CH_4_ production per unit volatile solids (in mL CH_4_/g VS); different pretreatment time was tested in order to study the effect of NaOH-1 N on enhancing the biomethane production of hydrolyses and degradation of lignocellulose. As shown in Figures 2(a)–2(e) and Table 4, the filter mud and bagasse pretreated by alkaline had significantly increased their cumulative biomethane yields in all codigestion ratios (*P* < 0.0001) compared with untreated ones, by increasing the cooking time of the substrate with NaOH. The highest cumulative biomethane production achieved in this study was 195.8 mL gVS^−1^ in digestion of pure bagasse treated for 45 min, which was 61.2% higher than untreated bagasse. However, an improvement of 66.6% was achieved in digestion of pure filter mud treated in the same cooking period (18.3 mL gVS^−1^) compared with untreated filter mud (6.1 mL gVS^−1^). These findings are consistent with previous studies [18, 45, 46] which verified the effectiveness of NaOH pretreatment in improving biodegradability and enhancing biomethane production. The cumulative biomethane yield was 92.8 mL gVS^−1^ when filter mud and bagasse were mixed in 50 : 50 ratio. The yield was decreased to 72.1 mL gVS^−1^ when filter mud content was increased to a mixture of 75 : 25 ratio. However, in all mixtures, the cumulative gas yield was lower as compared to that of bagasse alone, as the filter mud ratio increased in codigestion, the biomethane decreased drastically. Furthermore, the cumulative biomethane yield reached 126.2 mL gVS^−1^ when filter mud codigested with bagasse in 25 : 75 ratio increased by 81.20% from untreated composition.

### 3.4. Effect of pH

**Figure 3:**
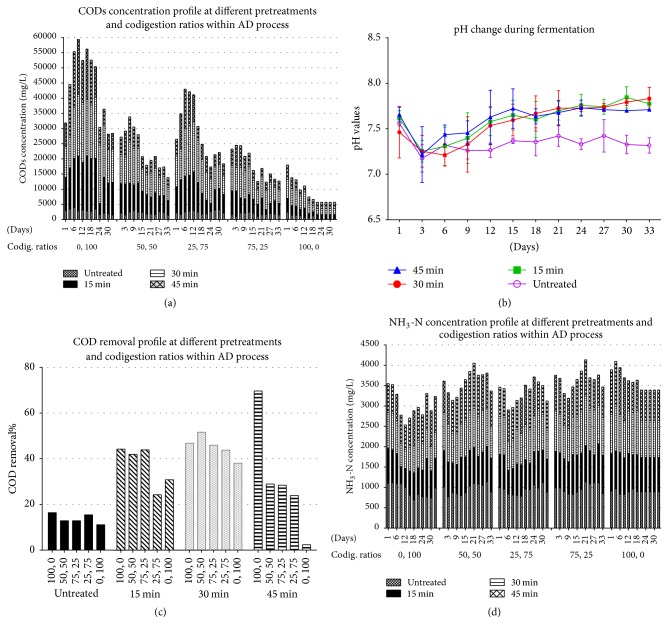
Basic anaerobic digestion process profiles.

Initially the pH of the substrate was found to be higher than 10 due to alkaline pretreatment. It was adjusted one time before starting fermentation in the range of 6.9–8.1 by the addition of hydrochloric acid (HCl); these values are in agreement with the operational range of 6.5–8.5 reported previously for an anaerobic digestion process [38]. The variation in pH over the period of digestion was within the range as shown in Figure 3(b). There was an initial decrease in pH values after two days of digestion as the concentration of acids increased, due to an imbalance between production and consumption of acids by methanogenic bacteria [47].

### 3.5. Effect of Pretreatment on CODs Concentration and COD Removal

Soluble chemical oxygen demand (COD) is considered the most important parameter for the anaerobic digestion process; the CODs were examined in three-day intervals during the anaerobic digestion process; NaOH pretreatment showed significant increase in the CODs concentration in all levels of codigestion, as shown in Figure 3(a). CODs values obtained after pretreatment were ranged from 1,312 to 20,384 mg/L in comparison with 320 to 3,912 mg/L for the untreated ones. At the beginning of the fermentation process, due to intense mineralization of the reactants, a considerable decrease in COD occurred. By the end, when microorganisms do not exhibit a living behavior and the process stopped, the CODs decreased; these results are in accordance with the previous research [44]. For untreated substrate, the CODs concentration increased rapidly when the percentage of bagasse increased in codigestion, which was 3,912; 3,065; 3,200; 2,480; and 2,416 mg/L for pure bagasse, 50% bagasse 50% filter mud, 75% bagasse 25% filter mud, 25% bagasse 75% filter mud, and pure filter mud, respectively. The maximum CODs value for treated bagasse and filter mud was obtained when bagasse and filter mud were treated for 30 min with NaOH, 20,384; 13,424; 16,448; 9,872, and 6,248 mg/L, respectively, with the same sequence mentioned above for untreated substrate. Moreover, CODs by 45 min of cooking were higher than 15 min. The highest COD solubilization reached in the current work was 20,384 mg/L obtained for reactor with pure bagasse pretreated with NaOH for 30 min, resulting in an increase of 80.8% with respect to untreated reactor with the same composition 3,912 mg/L. Meanwhile, the maximum CODs for reactor with pure filter mud pretreated with NaOH for 30 min were 6,248 mg/L, by increasing 60.3% with respect to untreated reactor with the same composition 2,480 mg/L. A possible reason for these higher CODs values is the alkaline pretreatment which causes hemicelluloses and parts of lignin to solubilize and subsequently higher organic degradation [40]. These results proved that pretreatment was more efficient with respect to promoting hydrolysis and increasing COD concentration. The highest COD solubilizations were achieved in the thermoalkaline pretreatments of sorghum forage and wheat straw, at 40 and 100°C with 10% NaOH for both substrates (around 30–40% for both substrates) [18]. González et al. evaluated different pretreatment time and lime loading on filter mud and reported that the highest COD solubilization was obtained when 3.18 g/L of Ca(OH)_2_ with 7.33 h of pretreatment time gives 6% over untreated press mud [12].

COD removal fluctuated in the initial startup stage of fermentation and stabilized after one week of operation. COD reduction was between 69.64 and 4% during the entire period of the study as shown in Figure 3(c). As could be shown, the percentage of COD removal values of the pretreated samples was significantly higher than the untreated sample. However, the highest methane yield (195.8 mL gVS^−1^) was observed in pretreated samples with the highest COD removal (69.64%).

### 3.6. Ammonia Nitrogen Concentration (NH_3_-N)

Nitrogen supplementation in the form of organic nitrogen or ammonia nitrogen is an essential nutrient for anaerobic digestion. It may inhibit microbial activities during anaerobic digestion process if it is available at high concentrations [48]. Inhibition of the anaerobic digestion process is usually indicated by the decrease in the steady state methane production rates and the increase in the intermediate digestion products like acids concentrations [49]. Such an unstable state may happen as a result of total ammonia nitrogen levels up to a range of 1500–7000 mg/L [50]. Currently, there are no reports about ammonia inhibitory effects on anaerobic digestion of lignocelluloses biomass with nitrogen supplementation. Wang et al. [51] have experimentally investigated the effect of total ammonia nitrogen on solid-state anaerobic digestion of corn stover. They found that concentrations higher than 2.5 g/kg (based on total weight) caused a significant reduction of methane yields by 50%. In this study, the concentration of NH_3_-N in all codigestion ratios and pretreatment levels was lower than level of inhibition as shown in Figure 3(d). Whereas for untreated substrate, the NH_3_-N concentration between 822 and 1,114 mg/L, pretreatment resulted in a decrease in the NH_3_-N concentration from 608.8 to 1,058 mg/L in the reaction with pure bagasse treated for 45 min.

## 4. Conclusions

The biomethane production from codigestion of filter mud and sugarcane bagasse with and without NaOH pretreatment was determined using anaerobic batch digesters. Lower C/N ratio (9.862) is the main problem of press mud for biomethanation. Mixing of other substrates of high C/N ratio like bagasse (26.646) resulted in optimum C/N ratio. Pretreatment by cooking the substrates at 100°C with NaOH 1 N for 45 minutes has a higher cumulative biomethane yield in all levels of codigestion compared to other pretreatment times and to untreated groups. Mixing of filter mud in the ratio of 25 : 75 (C/N ration 24.70) provided the best cumulative biomethane for untreated substrates and 126.2 mL gVS^−1^ with an increase of 81.20% from untreated composition for pretreated substrates. Furthermore, cellulose level increased after pretreatment which is in consistency with the decreasing level of lignin; the lignin content after cooking for 45 minutes was the lowest (3.096%). The results showed that codigestion with bagasse could be considered as an efficient method to improve biogas production from filter mud.

## Figures and Tables

**Table 1:** Bagasse and filter mud mixing ratio.

ID	Filter mud	Bagasse	C/N ratio

1	0%	100%	29.646
2	50%	50%	19.754
3	25%	75%	24.700
4	75%	25%	14.808
5	100%	0%	9.862

**Table 2:** Characteristics of filter mud and bagasse samples.

Parameters	Unit	Bagasse	Press mud
Total solids (TS)	(%)	58.900 ± 0.001	47.850 ± 0.002
Volatile matter (VS)	(%)	97.590 ± 0.003	34.780 ± 0.013
Organic matter	(%)	94.910 ± 3.086	28.150 ± 0.326
TOC	(%)	55.050 ± 1.789	16.330 ± 0.783
Total-N	(%)	1.857 ± 0.419	1.686 ± 0.325
Total-P	(%)	0.345 ± 0.052	nd^*∗*^
Total-K	(%)	2.100 ± 0.141	nd
C/N ratio		29.646	9.682

Note: each value represents mean ± STDEV of two replications.

nd^*∗*^ = not detected.

**Table 3:** Percentage of chemical compounds of sugarcane bagasse.

Treatment	Autoclaving time (minutes)	Hemicellulose (%)	Cellulose (%)	Lignin (%)
Untreated	0	32.293 ± 0.491	35.612 ± 2.775	22.563 ± 2.933
NaOH 1 N	15	11.258 ± 0.459	42.657 ± 1.851	4.514 ± 1.567
NaOH 1 N	30	8.732 ± 0.077	40.062 ± 0.029	4.715 ± 1.877
NaOH 1 N	45	11.083 ± 0.915	58.548 ± 0.014	3.096 ± 0.366

**Table 4:** Cumulative biomethane yields (in mL gVS^−1^) at different pretreatment periods and codigestion ratios.

Codigestion ratios	Pretreatment periods			

Filter mud, bagasse	0 min (untreated)	15 min	30 min	45 min
0%, 100%	76.0	97	113.0	195.8
50%, 50%	21.1	58.2	80.4	92.8
25%, 75%	23.7	79.9	96.4	126.2
75%, 25%	22.2	32.1	44.5	72.1
100%, 0%	6.1	17.5	9.2	18.3
